# PyRod Enables Rational Homology Model‐based Virtual Screening Against MCHR1

**DOI:** 10.1002/minf.202000020

**Published:** 2020-04-29

**Authors:** David Schaller, Gerhard Wolber

**Affiliations:** ^1^ Pharmaceutical and Medicinal Chemistry Freie Universität Berlin Königin-Luise-Strasse 2+4 14195 Berlin Germany

**Keywords:** MCHR1, PyRod, 3D pharmacophore, homology modeling, MD simulation

## Abstract

Several encouraging pre‐clinical results highlight the melanin‐concentrating hormone receptor 1 (MCHR1) as promising target for anti‐obesity drug development. Currently however, experimentally resolved structures of MCHR1 are not available, which complicates rational drug design campaigns. In this study, we aimed at developing accurate, homologymodel‐based 3D pharmacophores against MCHR1. We show that traditional approaches involving docking of known active small molecules are hindered by the flexibility of binding pocket residues. Instead, we derived three‐dimensional pharmacophores from molecular dynamics simulations by employing our novel open‐source software PyRod. In a retrospective evaluation, the generated 3D pharmacophores were highly predictive returning up to 35 % of active molecules and showing an early enrichment (EF1) of up to 27.6. Furthermore, PyRod pharmacophores demonstrate higher sensitivity than ligand‐based pharmacophores and deliver structural insights, which are key to rational lead optimization.

## Graphical abstract

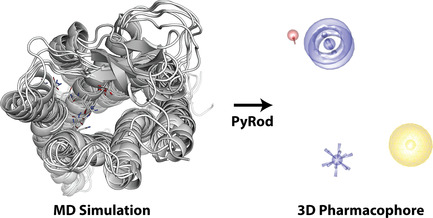

Obesity and overweight have progressed into major threats for human health causing four million deaths in 2015.^1^ Beside bariatric surgery that is associated with several complications,^2^ pharmaceutical intervention in combination with lifestyle intervention proved to be the most promising treatment option for obesity.^3^, ^4^, ^5^ However, currently approved anti‐obesity agents lack efficacy and show severe or unpleasant side effects.^6^

The melanin‐concentrating hormone receptor 1 (MCHR1) is a well characterized target for potential obesity treatment. Several rodent models of obesity showed encouraging results in knock‐out experiments or in administration of MCHR1 antagonists. Unfortunately, these promising results could not be translated to human obesity treatment, since all investigated drug candidates either raised safety concerns or were ineffective in clinical studies.^7^ However, there is evidence that the simultaneous antagonism of MCHR1 and histamine H_3_ receptor (H_3_R) might result in a synergistic effect that could be beneficial in obesity treatment.^8^ Also, we recently found three ligands that bind both receptors in the nanomolar activity range validating this target pair for rational multi‐target drug design campaigns.^9^

Structure‐based virtual screening campaigns employing atomistic models of the macromolecular target can be advantageous over ligand‐based campaigns, since hits confirmed by in‐vitro assays come with a potential binding hypothesis that can be exploited in subsequent lead optimization campaigns.^10^ Especially multi‐target drug design campaigns benefit from structural data, since lead molecules need to be optimized against multiple targets. Although the number of entries in the Protein Data Bank (PDB) is constantly increasing, many potential drug targets as well as validated drug targets still lack an experimentally resolved atomistic model.^11^ In such situation, researchers often employ homology modeling, a method that is generating an atomistic model of the target of interest based on a closely related macromolecule.^12^ However, performing structure‐based virtual screening using homology models increases the chance for modeling artifacts, since even small modeling errors, such as a wrong side chain conformation essential for ligand binding, can impair docking performance.^13^, ^14^

Molecular dynamics (MD) simulation can be used to address such artifacts and additionally, provide valuable information about the flexibility and thermodynamic properties of the system.^15^, ^16^, ^17^, ^18^, ^19^ PyRod, a free and open‐source Python software, combines the strength of MD simulations with structure‐based 3D pharmacophore searches by analyzing the protein environment of water molecules in protein binding pockets and subsequently generates pharmacophore features for virtual screening.^20^

In this study, we aimed at generating highly predictive structure‐based 3D pharmacophore models for virtual screening against MCHR1. A highly flexible hydrogen bond network involving three glutamine residues in the binding pocket of MCHR1 hindered the use of conventional workflows employing docking algorithms for pose prediction. Hence, we applied our software PyRod that analyzes the protein environment of water molecules in protein binding pockets throughout an MD simulation for pharmacophore feature placement. The presented workflow (Figure 1) yielded 3D pharmacophores that were highly successful in discriminating actives from decoys in a retrospective virtual screening campaign. Furthermore, they provide structural insights for binding to MCHR1 that are not obtainable by ligand‐based pharmacophore modeling.

**Figure 1:**
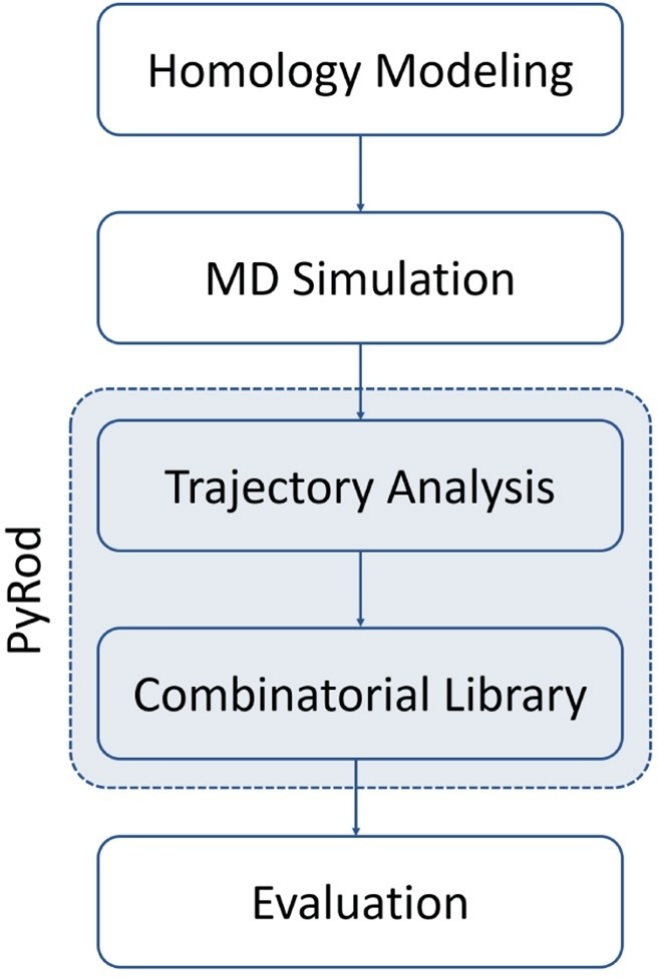
Workflow diagram for generating homology model‐based 3D pharmacophores against MCHR1 with PyRod.

A sequence search identified crystal structure 4N6H^21^ of the inactive δ opioid receptor to be a suitable template for homology modeling of MCHR1 with a sequence similarity of 50.2 %. A homology model of MCHR1 in the inactive state was generated with MOE 2018.^22^ The sodium ion complexed by D^2.50^ was transferred from 4N6H into the MCHR1 homology model, since it was found to be structurally important for the inactive state of class A GPCRs.^23^ A ramachandran plot analysis in MOE 2018 underlined the quality of the model with 95 % of dihedral angles located in the core region, 5 % in the allowed region and none outside.

The orthosteric pocket residues Q^3.36^, Q^5.42^ and Q^6.55^ are highly flexible allowing various conformations and interact with several neighboring residues in a complex hydrogen bonding network (Figure 2). Such situations complicate docking studies, since their performance can already be affected by small changes in side chain conformations.^13^, ^14^ Thus, this homology model was subjected to molecular dynamics simulations with Desmond 5.1^24^ to explore side chain conformations. The trajectories were analyzed using PyRod 0.7.2^20^ to identify potential hotspots for ligand binding and to generate 3D pharmacophores for virtual screening. The simulation time of the 10 replicates was increased from 10 ns to 30 ns compared to the original PyRod publication to relax artifacts introduced through homology modeling.^20^ The last 10 ns of each replicate were analyzed with PyRod granting sufficient sampling of side chain conformations.^25^, ^26^

**Figure 2:**
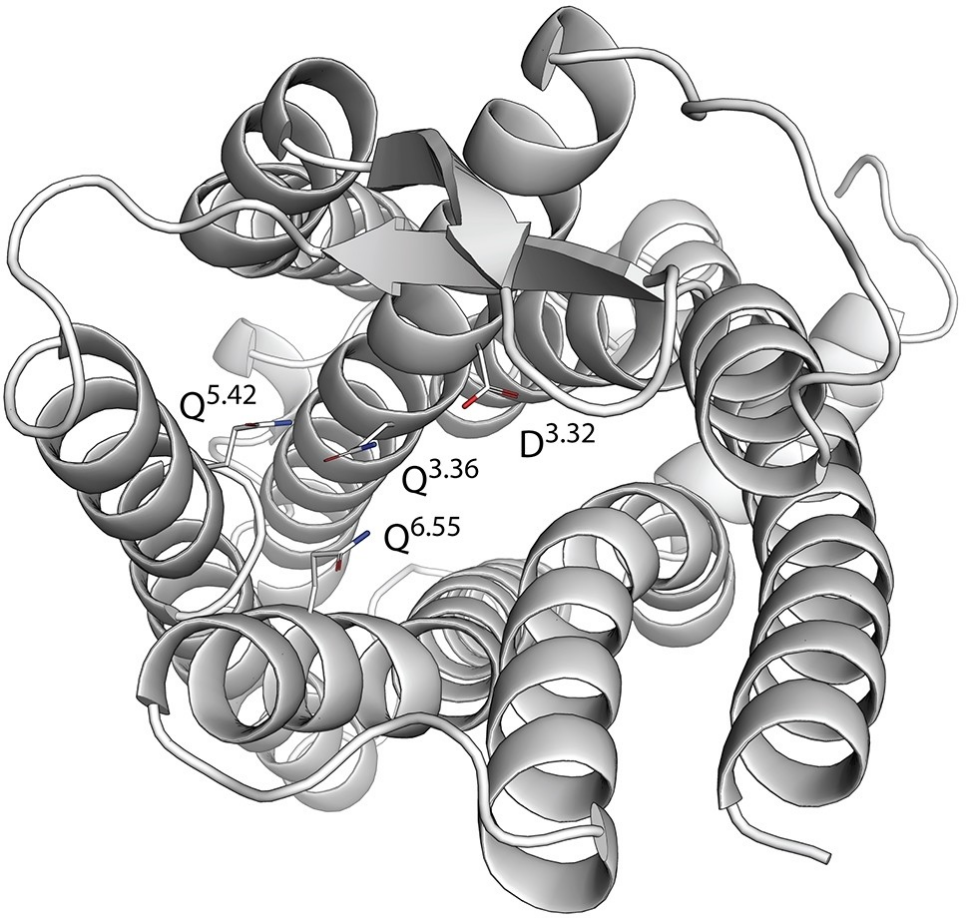
Top view into the binding pocket of the MCHR1 homology modeling. The three flexible glutamines can adapt various conformations.

The PyRod software describes pharmacophoric binding pocket characteristics in form of dynamic molecular interaction fields (dMIFs) for common pharmacophore features including hydrogen bonds, ionizable and aromatic interactions as well as hydrophobic contacts. PyRod suggests favorable regions for hydrogen bonding and charged interactions close to D^3.32^ and the sodium ion complexed by D^2.50^ (Figure 3A). Additional hotspots for hydrogen bonding are located next to Q^3.36^, Q^5.42^ and Q^6.55^ supporting our hypothesis on the potential participation of these residues for ligand binding. Several hydrophobic residues are present in the orthosteric binding pocket favoring hydrophobic contacts above D^3.32^, next to the sodium ion and close to the glutamines 3.36, 5.42 and 6.55 (Figure 3B). Sites for possible aromatic interactions can be found between residues W^6.48^ and F^2.53^ next to the sodium ion and in the upper part of the binding pocket next to extracellular loop residues R353 and F256.

**Figure 3:**
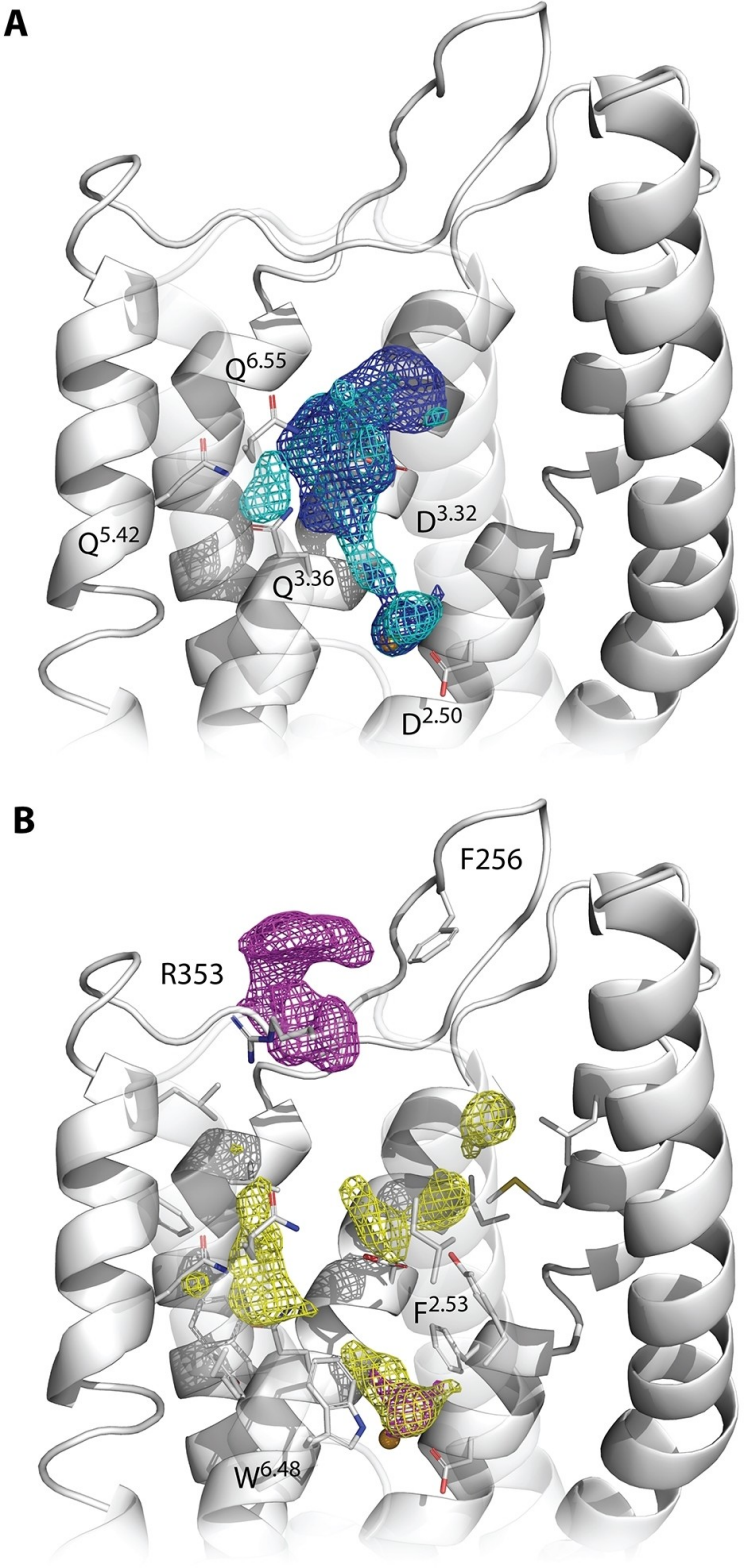
PyRod analysis of MCHR1 orthosteric binding pocket. Depicted dMIFs represent favorable regions for (A) hydrogen bonding (cyan, cutoff=27.8) and positive ionizable interactions (blue, cutoff=25.3) as well as for (B) hydrophobic (yellow, cutoff=111.8) and aromatic features (magenta, cutoff=15.3). Cutoffs were chosen based on the half maximum of the respective feature class. Transmembrane helices 6 and 7 were set transparent to allow better visualization.

PyRod generates a single super‐pharmacophore containing all possible interaction points by analyzing dMIFs for each pharmacophore feature type. However, this 3D pharmacophore consists of too many features for efficient virtual screening. Thus, prioritization and selection of pharmacophore features is mandatory for further processing and was performed by analyzing dMIFs for each respective pharmacophore feature type manually. Hotspots for interactions close to the sodium ion were ignored, since, to our knowledge, no ligands of GPCRs were reported to replace or interact with this sodium ion. Pharmacophore features were selected to show a high PyRod score according to the respective dMIF and to cover the orthosteric binding pocket. Additionally, pharmacophore features were included that are located close to extracellular loops, since these regions were found to frequently contribute to ligand binding.^27^ The focused 3D pharmacophore model consists of 15 features (Figure 4A), i. e. two positive ionizable interaction features and two associated hydrogen bond donors pointing towards D^3.32^, four hydrogen bonding features close to glutamines 3.36, 5.42 and 6.55, two hydrogen bond acceptors close to the extracellular loops of MCHR1, four hydrophobic features covering both pockets next to the three glutamines and above D^3.32^, and one aromatic feature next to R353. Notably, PyRod aggregates feature frequency with geometric criteria in an aggregating scoring function delivering interaction hotspots; therefore, there is no guarantee for the simultaneous occurrence of features in a single frame.

**Figure 4:**
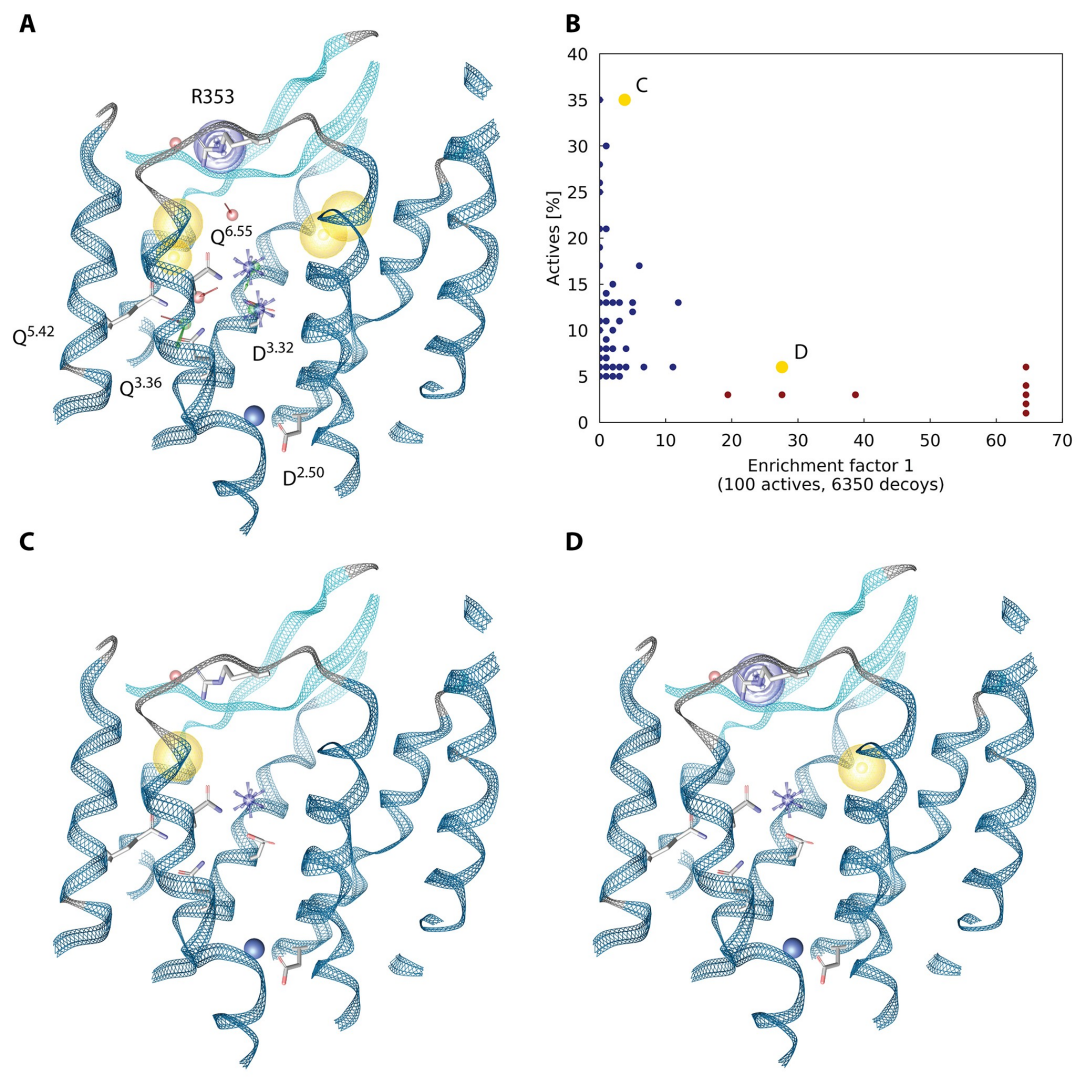
3D pharmacophores and their performance. (A) Focused 3D pharmacophore from PyRod used for combinatorial processing (for scores of selected pharmacophore features see supporting information Tab S1). (B) Evaluation of 3D pharmacophores against a MCHR1 test set. The blue and yellow dots represent the performance of PyRod pharmacophores, red dots represent the performance of ligand‐based shared feature pharmacophores generated with LigandScout 4.2. (C, D) 3D pharmacophores identifying the most actives from MCHR1 test set and showing the highest early enrichment respectively. Exclusion volumes were not depicted for the sake of clarity. Blue star‐positive ionizable, yellow sphere–hydrophobic contact, purple ring‐aromatic interaction, red arrow‐hydrogen bond acceptor.

The focused 3D pharmacophore was subjected to combinatorial processing with PyRod 0.7.2.^20^ Feature combinations were restricted to 3D pharmacophores with minimal 3 and maximal 5 spatially independent chemical features to reduce the combinatorial space. Additionally, each 3D pharmacophore must contain one positive ionizable feature to further limit combinatorial space and to focus on ligands carrying a positive charge, which would be beneficial for potential binding to H_3_R. This procedure resulted in 1136 different 3D pharmacophores against MCHR1. 3D pharmacophores were evaluated with LigandScout 4.2^28^ for discrimination of a diverse set of 100 actives retrieved from the ChEMBL 24 database^29^ and 6350 matched decoys from the DUD‐E server.^30^

Altogether, 62 3D pharmacophores were able to retrieve at least 5 % of the MCHR1 active set, which was the criteria to advance to the computationally more expensive decoy screening (Figure 4B). The results from actives and decoys screening were used for calculation of the early enrichment factor (EF_1 %_). The 3D pharmacophore with the highest true positive hit rate was able to identify 35 % of the actives and consists of one positive ionizable feature next to D^3.32^, one hydrophobic feature above the three glutamines 3.36, 5.42 and 6.55, as well as one hydrogen bond acceptor feature close to the extracellular loops (Figure 4C, supporting information Figure S1A). However, this 3D pharmacophore only achieves considerably weak early enrichment (EF_1 %_=4.0). In contrast, the 3D pharmacophore with the best enrichment (EF_1 %_=27.6) also carries an aromatic feature next to R353 and additionally has the hydrophobic feature located above D^3.32^, but only picks 6 % of the active molecules (Figure 4D, supporting information Figure S1B). Further analysis of the hit lists revealed that the two described 3D pharmacophore models only share two active hits. Thus, they can be considered complementary leading to improved performance when screened as ensemble.

A more simple alternative approach would be the generation of 3D pharmacophores from alignments of known active molecules. Hence, we were interested if PyRod pharmacophores can achieve a similar performance compared to ligand‐based pharmacophores. The complete active set, containing 695 unique MCHR1 ligands, was clustered with LigandScout 4.2^28^ resulting in 19 clusters comprising of at least 10 molecules. Each of these clusters was employed to generate a shared‐feature pharmacophore. In total, 12 pharmacophores contained the important positive ionizable feature and were evaluated for early enrichment and retrieval of known actives (Figure 4B, supporting information Figure S1C). All tested ligand‐based shared‐feature pharmacophores show very high early enrichment factors of up to 64.5. However, the ligand‐based pharmacophores are not as sensitive returning at most 6 % of actives from the test set. Furthermore, such 3D pharmacophores lack any information about the interactions with the receptor, which is essential to rational lead optimization.

This is the first study applying PyRod on MD simulations of a homology model. By employing PyRod, we were able to generate several 3D pharmacophores against MCHR1 that are highly successful in discriminating active MCHR1 ligands from decoys. The 3D pharmacophore generation is thereby not dependent on error‐prone docking studies in homology models but instead exploits water dynamics from MD simulations. Hits identified with these structure‐based 3D pharmacophores hold the information of a binding hypothesis that can be used for subsequent rational lead optimization. Furthermore, we show that PyRod pharmacophores present an attractive alternative to ligand‐based pharmacophores that heavily dependent on correct ligand conformations as well as their alignment, and additionally, lack information essential for further lead optimization.^31^ These characteristics render PyRod pharmacophores highly valuable tools for hit identification and optimization in anti‐obesity drug design campaigns against MCHR1. Also, the presented workflow (Figure 1) can be easily transferred to other projects that aim at performing homology model‐based virtual screening campaigns.

## Experimental Section

A template search using the GPCRdb^32^ and subsequent analysis in MOE 2018^22^ revealed the high resolution crystallographic structure 4N6H^21^ of the δ opioid receptor as suitable template for generating a homology model of MCHR1. The amino acid sequence of human MCHR1 (Q99705) was retrieved from Uniprot^33^ and aligned to the crystallographic structure 4N6H in MOE 2018 according to the proposed alignment from GPCRdb (supporting information Figure S2). The aligned sequences show a sequence identity of 29.4 % and a sequence similarity of 50.2 %.

Employing this alignment, a homology model of human MCHR1 was generated based on 10 main chain models with 10 side chain samples per main chain model at 300 K in MOE 2018. Automatic model refinement was disabled. The structurally important sodium ion and 5 water molecules were transferred from the template structure 4N6H. The side chain conformation of S195 was refined to allow correct complexation of the sodium ion (supporting information Figure S3). Atom clashes were sequentially minimized with OPLS‐AA force field^34^ implemented in MOE 2018. Protonation was performed using the Protonate3D tool in MOE 2018.

The homology model of MCHR1 was subjected to molecular dynamics simulation. Chain breaks were capped with NME and ACE in MOE 2018.^22^ The receptor was oriented using the PPM server^35^ for subsequent membrane placement in a POPC bilayer using Maestro 11.3^36^ and solvation in a orthorhombic box of TIP4P water with 10 Å padding containing 0.15 M NaCl. In total, 10 replica of 30 ns MD simulations were performed using Desmond 5.1.^24^ Frames were saved every 10 ps resulting in 3000 frames per simulation. The pbc wrap functionality implemented in VMD 1.9.3^37^ was employed to center the receptor in the periodic boundary box and the RMSD Trajectory Tool to align the trajectory on the heavy atoms of the protein backbone of the first frame.

The test grid component of PyRod 0.7.2^20^ was used to identify appropriate parameters for grid placement. The identified parameters result in cubic grids with an edge length of 30 Å spanning the orthosteric binding pocket of MCHR1 (supporting information Figure S4). The last 10 ns of each simulation were analyzed using the trajectory pharmacophore combo of PyRod 0.7.2 with default parameters resulting in the generation of dynamic molecular interaction fields describing pharmacophoric binding pocket characteristics as well as a super pharmacophore describing potential interaction sites with the receptor.

The CHEMBL 24 database^29^ was used to retrieve activity data for MCHR1 (CHEMBL344). Ligands were filtered for molecular weight (≤700), confidence score (≥9), standard relation (=), standard value (≤10) standard units (nM) and standard type (K_i_, K_d_, IC_50_ or EC_50_). RDKit^38^ nodes implemented in KNIME 3.7.1^39^ were used to remove molecules with unspecified stereo centers and to remove duplicates, whereas binding data was preferred over functional data and more recent data points were preferred over older. This procedure resulted in 695 unique ligands of MCHR1.

MOE 2018^22^ was used to identify the dominant protonation state at pH 7 and Corina 3.00^40^ to generate a low‐energy 3D conformation. The RDKit diversity picker was employed in KNIME 3.7.1 to pick 100 diverse active ligands (for a distribution of activity values see supporting information Figure S5). The DUD‐E server^30^ was used to generate decoys for the selected diverse ligands. In total, 6350 decoys were retrieved from DUD‐E server, protonated at pH 7 in MOE 2018 and an initial conformation was generated with Corina 3.00. By employing iCon implemented in idbgen from LigandScout 4.2^28^ 25 conformations were generated for each of the molecules in the active and decoy sets for later 3D pharmacophore evaluation.

LigandScout 4.2 was employed to visualize and analyze the previously generated dMIFs guiding the selection of pharmacophore features from the super pharmacophore for combinatorial library generation with PyRod 0.7.2.^20^ Fifteen features were selected and combined to 1136 different 3D pharmacophores. The combinatorial space was limited by restricting 3D pharmacophores to contain 3–5 independent features, 1–3 hydrogen bonds, 0–1 aromatic interaction and exactly 1 ionizable interaction. Each 3D pharmacophore was evaluated with LigandScout 4.2 for discrimination of actives from decoys which were generated as already described.

Ligand‐based shared‐feature pharmacophores were generated in LigandScout 4.2. All 695 unique MCHR1 ligands were clustered and clusters comprising of at least 10 molecules were subjected to shared‐feature pharmacophore generation. 3D pharmacophores containing a positive ionizable feature were evaluated for early enrichment factor and retrieval of actives as already described.

## Conflict of Interest

None declared.

## Supporting information

As a service to our authors and readers, this journal provides supporting information supplied by the authors. Such materials are peer reviewed and may be re‐organized for online delivery, but are not copy‐edited or typeset. Technical support issues arising from supporting information (other than missing files) should be addressed to the authors.

Supplementary
