# Supplementary material for: PyRod Enables Rational Homology Model‐based Virtual Screening Against MCHR1
Source: Mol Inform. 2020 Apr 29;39(6):2000020. doi: 10.1002/minf.202000020 (PMC7317519; doi:10.1002/minf.202000020)
Supplement: Supplementary file 1 — Supplementary [file MINF-39-2000020-s001.pdf]

molecular informatics

Supporting Information

PyRod Enables Rational Homology Model-based Virtual Screening Against MCHR1

David Schaller and Gerhard Wolber*© 2020 The Authors. Published by Wiley-VCH Verlag GmbH & Co. KGaA. This is an open access article under the terms of the Creative Commons Attribution License, which permits use, distribution and reproduction in any medium, provided the original work is properly cited.

Supporting Information

PyRod Enables Rational Homology Model-Based Virtual Screening Against MCHR1

David Schaller[a] and Gerhard Wolber*[a]

[a] Pharmaceutical and Medicinal Chemistry, Freie Universität Berlin, Königin-Luise-Straße 2+4, 14195 Berlin, Germany

*e-mail: gerhard.wolber@fu-berlin.de, phone: +49 30 83852686

Table of contents

1. **Feature Scores** 2
2. **ROC Statistics** 3
3. **Sequence Alignment** 4
4. **Optimization of Sodium Coordination** 5
5. **Grid Placement for PyRod Analysis** 6
6. **Distribution of Activity Values** 7

1. Feature Scores

Table S1: Scores for selected pharmacophore features according to the respective dMIF generated by PyRod. Feature 11 and 12 are part of a mixed hydrogen bond donor/acceptor feature and received the same score. H – hydrophobic contact, PI – positive ionizable, AR – aromatic interaction, HBA – hydrogen bond acceptor, HBD – hydrogen bond donor.

	Feature Type	Feature Score
1	H	105.35
2	H	122.83
3	H	114.85
4	H	154.07
5	PI	42.76
6	PI	34.07
7	AR	17.83
8	HBA	16.24
9	HBA	14.95
10	HBA	14.8
11	HBA	11.71
12	HBD	11.71
13	HBD	36.07
14	HBD	28.19
15	HBD	22.9

2. ROC Statistics

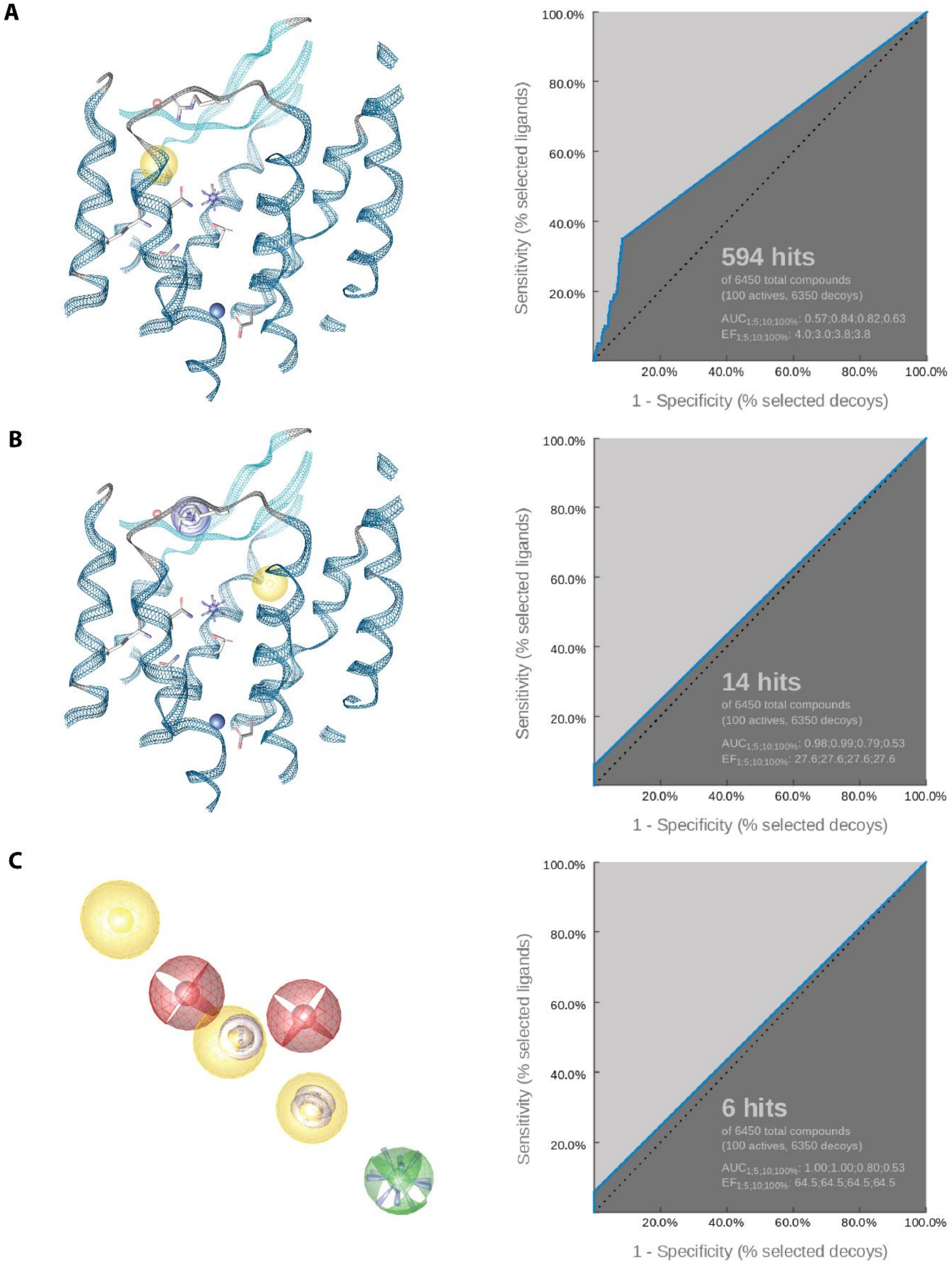

Figure S1: ROC statistics for selected 3D pharmacophores generated with PyRod (A, B) and with the ligand-based mode in LigandScout 4.2 (C). Exclusion volumes are not depicted for the sake of clarity. Blue star – positive ionizable, yellow sphere – hydrophobic contact, purple ring – aromatic interaction, red arrow and sphere – hydrogen bond acceptor, green sphere - hydrogen bond donor.

3. Sequence Alignment

```
              <----------------H1---------------->         <-------------
MCHR1        PRTGSISYINIIMPSVFGTICLLGIIGNSTVIFAVVKKSKLHWCNNVPDIFIINLSVVDL
DOR (4N6H)   ARSASSLALAIAITALYSAVCAVGLLGNVLVMFGIVRYTKM---KTATNIYIFNLALADA
              *:.*   : * : :::.::* :*::**  *:*.:*: :*:    .. :*:*:**: :.*
                                                  <-ICL1-->

             H2------------>       <---------------H3--------------->
MCHR1        LFLLGMPFMIHQLMGNGVWHFGETMCTLITAMDANSQFTSTYILTAMAIDRYLATVHPIS
DOR (4N6H)   LATSTLPFQSAKYLME-TWPFGELLCKAVLSIDYYNMFTSIFTLTMMSVDRYIAVCHPVK
             *    :**   : : : .* *** :*. : ::*  . *** : ** *::***:*..**:.
                            <ECL1->                                  <---

                   <-----------H4---------->                    <--------
MCHR1        STKFRKPSVATLVICLLWALSFISITPVWLYARLIPFPGGAVGCGIRLPNP-DTDLYWFT
DOR (4N6H)   ALDFRTPAKAKLINICIWVLASGVGVPIMVMAVTRPRD-GAVVCMLQFPSPSWYWDTVTK
             : .**.*: *.*:   :*.*:    .*: : *   *   *** * :::*.*
             ICL2->                         <-------ECL2------->

             --------H5----------------->       <------------------H6----
MCHR1        LYQFFLAFALPFVVITAAYVRILQRMTSSVAPASQRSIRLRTKRVTRTAIAICLVFFVCW
DOR (4N6H)   ICVFLFAFVVPILIITVCYGLMLLRLRSVRLLSGSKEKDRSLRRITRMVLVVVGAFVVCW
             :  *::**.:*:::**..*  :* *: *    :..:.     :*:** .:.:  .*.***
                                         <ICL3->

             ----------->       <------------H7------------> <----H8---->
MCHR1        APYYVLQLTQLS  ISRPTLTFVYLYNAAISLGYANSCLNPFVYIVLCETFRKRLVLSVK
DOR (4N6H)   APIHIFVIVWTLVDIDRRDPLVVAALHLCIALGYANSSLNPVLYAFLDENFKRCFRQLCR
             ** ::: :      *.*    .*   . .*:******.***.:* .* *.*:: :    .
                         <ECL3->

MCHR1        PAAQ
DOR (4N6H)   KPCG
```

Figure S2: The depicted sequence alignment was used for homology modeling of human MCHR1. The template sequence is highlighted according to structural properties. Red sections represent helices, yellow sections represent β-sheets and underlined cysteines are involved in a disulfide bond. Furthermore, the sequence alignment contains information about the naming of helices and loops as well as the sequence similarity. (H1) - helix 1, (ICL1) - intracellular loop 1, (ECL1) - extracellular loop 2, (*) - identical residues, (:) - residues with high similarity, (.) - residues with low similarity.

4. Optimization of Sodium Coordination

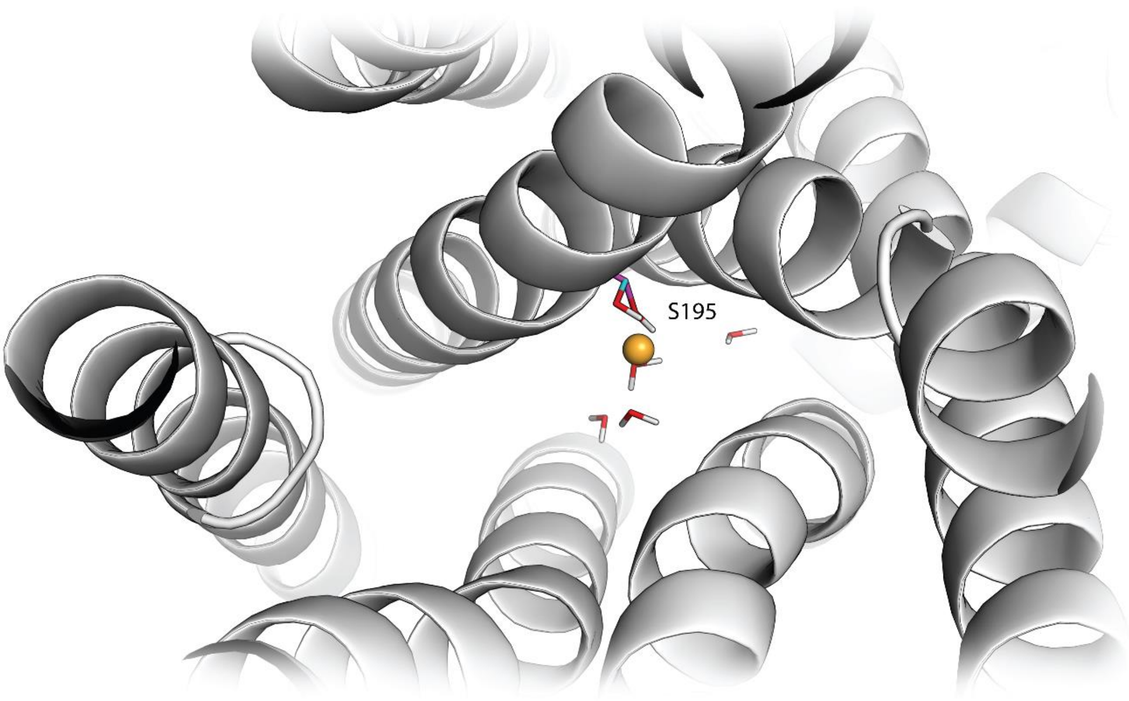

Figure S3: Refinement of the homology model of MCHR1. The conformation of S195 was altered to allow correct complexation of the important sodium ion highlighted in orange. Carbon atoms prior refinement are colored in cyan, carbon atoms after refinement in magenta.

5. Grid Placement for PyRod Analysis

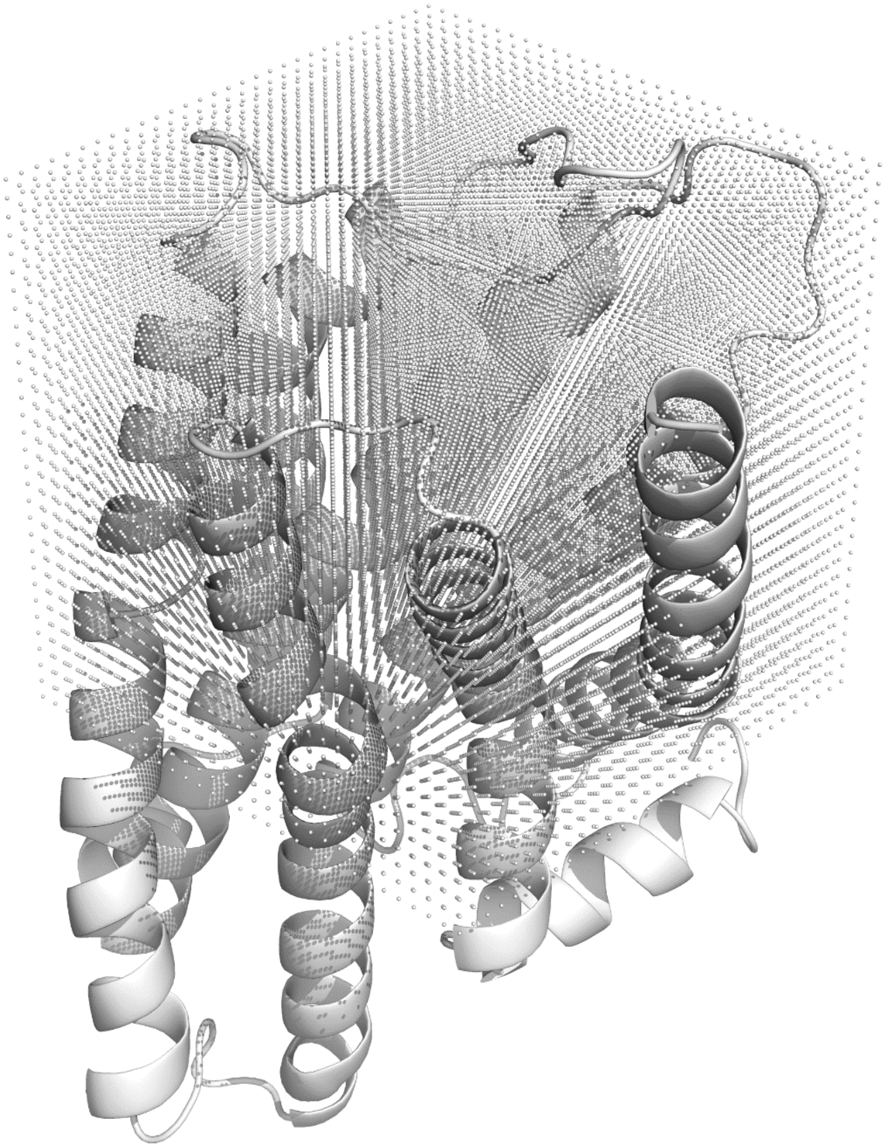

Figure S4: Test grid placement in the orthosteric binding pocket of MCHR1 for later PyRod analysis.

6. Distribution of Activity Values

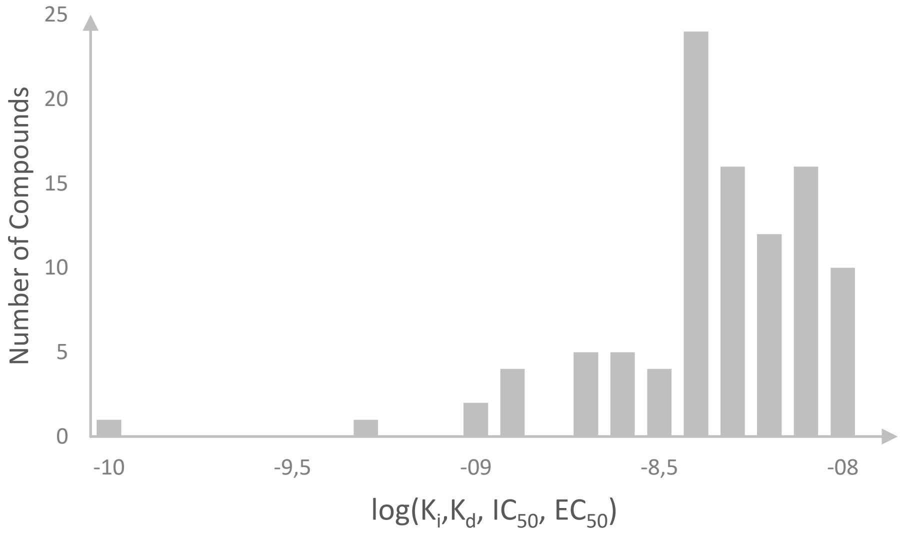

Figure S5: Distribution of activity values for the MCHR1 active set.
